# Registration and primary outcome reporting in behavioral health trials

**DOI:** 10.1186/s12874-021-01500-w

**Published:** 2022-02-06

**Authors:** Nicholas J. Taylor, Dennis M. Gorman

**Affiliations:** Department of Epidemiology and Biostatistics, Texas A&M University, College Station, TX USA

**Keywords:** Trial registration, Study protocols, Randomized trials, Selective outcome reporting, Replication, Behavioral health interventions

## Abstract

**Background:**

Registration of research studies is designed to lock investigators into a data collection and analysis plan before a study starts and thereby limit their ability to engage in flexible data analysis and selective outcome reporting. Studies of registered clinical trials show that one- to two-thirds are registered after the study has started and that non-adherence to important design and analytic features, such as reporting data pertaining to all primary outcomes, remains high. Less is known about the effects of registration on research transparency and integrity outside of clinical trials. To address this gap in knowledge, the current study examined the effects of registration on the reporting of research findings in a sample of behavioral health trials published in *BMC Public Health*.

**Methods:**

Registered trials published in the *BMC Public Health* section “Health Behavior, Health Promotion and Society” between 2011 and 2015 were included in the study. For each trial, we reviewed associated online submissions from 13 different registration sites. For those determined to have been prospectively registered, we used the trial registry, MEDLINE (Pubmed), PsychINFO, Web of Science and e-mails to investigators to identify subsequent publications from the study that reported results pertaining to primary outcomes. The two investigators then independently reviewed the outcome publication(s) and compared the primary outcomes reported in these to the registered primary outcomes.

**Results:**

The final analytic sample comprised 136 locatable, registered trials with an identifiable start date. Sixty-eight of the 136 were prospectively registered. Among these prospectively registered trials, only 16 published manuscripts reported outcomes and methods that were concordant with their registrations.

**Conclusions:**

Retrospective submission of protocols for publication and retrospective registration remain common in public health research, and adherence to prespecified outcomes is rare. In its current form, registration of behavioral and health promotion trials is likely to have minimal effect on preventing selective outcome reporting in publications, and the pervasiveness of vague and incomplete registry entries means that registries will have limited utility in terms of facilitating replication studies.

**Supplementary Information:**

The online version contains supplementary material available at 10.1186/s12874-021-01500-w.

## Registration

Protocol-Publication Comparison and p-curve Analysis in BMC Public Health (#34960), AsPredicted, Wharton Credibility Lab, University of Pennsylvania (see supplemental material).

## What is New

To our knowledge, this is the first study to examine registration-publication discordance in behavioral trials in public health; the study began by identifying registration submissions for randomized controlled trials and then proceeded to identify all published manuscripts describing results of those trials. Prior studies have not taken this approach, instead opting to start by identifying published manuscripts and working backwards to registration submissions. This study was also the first to examine published protocols in detail, and whether they were serving their intended purpose.

## Background

Pre-registration of study methods, including analytic plans, has been suggested as a deterrent to presenting exploratory (hypothesis generating) research as confirmatory (hypothesis testing) research in scholarly publications [1–3]. Such registration is designed to lock investigators into a data collection and analysis plan before a study begins, inhibiting the potential for flexible data analyses driven by confirmation bias [4].

Since 2004, the International Committee of Medical Journal Editors (ICMJE) has required that a manuscript describing the results of a clinical trial only be considered for publication if the trial is registered in a public registry prior to subject enrollment [5]; and, a similar requirement was introduced into the Declaration of Helsinki four years later [6]. These requirements have resulted in similar recommendations to authors from health-related academic journals [7–10].

Despite the widespread adoption of registration requirements by academic journals, especially those that publish clinical trials, its effects on the quality of outcome reporting have only recently begun to be critically evaluated. Some studies have shown inadequate adherence to important study design and analytic features such as primary outcomes, follow-up schedules, and prescribed statistical analyses [11–15]. Even when there is agreement between a registered protocol and the methods detailed in a published manuscript, confidence in reported results is undermined by the fact that one- to two-thirds of registered clinical trials are registered after the study began (retrospective registration) [16–18]. Adherence is even lower in observational research, with registration frequently taking place after the study has started and pre-specification of outcomes and data analyses occurring rarely [19].

Outside of medical research, registration of studies is uncommon; and among those studies that are registered, adherence to the registered protocol is poor [20–22]. Since we know of no previous studies that have examined the effects of registration on the reporting of research findings in public health, the current study was designed to address this gap in the public health literature. Moreover, we adopted a novel approach to evaluating concordance between registry protocols and journal publications. Previous studies traced a registration entry from a single published manuscript under the assumption that all primary outcomes indicated in a registration submission are reported in a single publication. This assumption is rather weak in the context of public health; it is common for investigators performing trials of behavioral intervention programs to examine multiple outcomes over extended follow-up periods, and to publish results in more than one manuscript. To address this limitation, the current study began with a published protocol that described a specific registered trial. From this registration data, explicit study commencement and ending dates could be ascertained, allowing us to distinguish prospective from retrospective studies. Subsequent publications reporting results from the prospectively registered trials were then identified.

*BMC Public Health* was specifically chosen for this investigation because, in its instructions to authors, the journal states, “By publishing your protocol in *BMC Public Health*, it becomes a fully citable open-access article. Publication of study protocols can reduce publication bias and improve reproducibility [23].” Thus, *BMC Public Health* clearly considers the publication of protocols as a means of reducing flexible data analysis and selective outcome reporting. Further, *BMC Public Health* advises “Protocols of randomized trials should follow the SPIRIT guidelines”, which state “ a well-written protocol facilitates an appropriate assessment of scientific, ethical, and safety issues *before a trial begins*; consistency and rigor of trial conduct; and full appraisal of the conduct and results after trial completion” [24].

## Methods

### Sample selection

The current study identified all published articles labeled as “protocols” in the *Health Behavior, Health Promotion and Society* section of *BMC Public Health* between 2011 and 2015. We restricted our study to these years to ensure a reasonable interval of time between protocol publication, completion of trial, and publication of trial results. For each study protocol, we recorded the date of receipt by *BMC Public Health*—a time point which precedes the journal’s actual publication date of the protocol. In light of the evidence showing low adherence to registration among observational studies, we further restricted consideration to those protocols that described registered randomized controlled trials (RRCTs).

### Registration data abstraction

For each RRCT described, we reviewed associated online registration submissions from 13 different trial registration sites and recorded the following data: trial registration number (if available), trial registration date (i.e. the earliest date of trial submission for registration, which may precede the official online registration publication date); trial start date (i.e. the date of first trial participant enrollment or the date of first data collection, whichever came first); trial end date (i.e. date of final data collection); the primary outcome(s) to be measured in the trial; and whether the primary outcome(s) had been amended subsequent to trial start date (where this information was available). Given the different fields/language used for each of the 13 registration sites, we evaluated each site and standardized fields to our study definitions of trial registration date, trial start date, trial end date, and primary outcome(s) (Supplemental Table 1 ). Full details of all data collected can be found in Supplemental Table 2 .

### Analytic cohort

Our primary analysis was limited to those RRCTs that were prospectively registered (i.e. those trials that were registered before the trial start date). One registration site (clinicaltrials.gov) only reports month/year of trial start date. To mitigate the potential biasing effects that could ensue from trials registering late in the same month as their trial’s start date, we judged as prospective any trial registered at clinicaltrials.gov in the first 10 days of the same month that trial is indicated to have started. If a trial registered at clinicaltrials.gov was registered and started in the same month, and registration was after the tenth day of the month, it was considered retrospective.

### Identification of published trial results

After finalizing the analytic cohort of RRCTs, we reviewed the trial registry for references to subsequent publications from the study reporting results pertaining to outcomes. Next, a systematic search of publications describing primary outcome results was performed for each RRCT using the following databases: MEDLINE (Pubmed); APA PsycINFO; and Web of Science. A defined set of search terms was used for each database, and included: Principal Investigator’s full name; trial registration number; unique trial name (where available); and key words relevant to individual RRCTs. The searches were conducted in June and July, 2020; a detailed accounting of search terms used in this study can be found in Supplemental Table 3 .

Once the publications reporting results for primary outcomes from each RRCT were identified, up to three letters were sent via e-mail to each of the principal investigators or lead contact of the prospectively registered trials asking them to review the list of publications identified. If no publications were identified through the database search, the principal investigator or lead contact was asked to confirm that there were no subsequent publications from the study.

### Analysis

Both investigators independently reviewed the outcome publication(s) for the RRCTs included in the analytic cohort and compared the primary outcome reported in these to the registered primary outcomes. The two investigators met and discussed their evaluations of the concordance between the registered primary outcomes and those reported in subsequent publications. The review and rating of each study focused on the description of the primary outcomes and how these were measured, as well as the timing of the follow-up assessment point(s). For the registries, we used the primary outcome and secondary outcome categories included in these to differentiate primary and secondary outcomes. For the publications, we used the authors’ categorization of variables as primary or secondary when they were specified; when they were not, and authors just reported outcome variables, we considered them all to be primary outcomes.

The specific focus of the review was on (a) the introduction of unregistered primary outcomes into the publication, (b) the promotion of registered secondary outcomes to primary outcomes in the publication, (c) failure to report on a registered primary outcome in the publication, (d) relegation of a registered primary outcome to a secondary outcome in the publication, and (e) failure to adhere to the registered follow-up assessment protocol in the publication. Additionally, trial registration submissions frequently contained vague descriptions of both the primary outcomes and the timing of follow-up assessments and often failed to specify how primary outcomes would be assessed/measured. Such vague registry descriptions of primary outcomes, measures and assessment periods made it difficult to ascertain whether these were congruent with those reported in the publications; indeed, a vague description with no measure specified could be congruent with a large number of outcomes and measures described in a paper. Accordingly, the studies were also rated in terms of (f) whether the registry description of the primary variables was vague, (g) whether the measures of the primary variables were specified, and (h) whether the follow-up assessment protocol was well-defined.

### Changes from registered protocol

This study was registered in the AsPredicted registry before data collection began. The registry entry, which can be found in Supplementary Materials, proposed two phases: Phase 1 (Aim 1)—to examine whether there is consistency between the information contained in published protocols pertaining to behavioral and social science public health research and subsequent publications from these research projects; Phase 2 (Aim 2)—to distinguish two groups of published manuscripts identified in Phase 1: (a) those that explicitly followed their associated protocols and (b) those that did not. The current paper pertains to Aim 1. We proposed, in the registry, to review 51 protocols published in *BMC Public Health* in 2011 and 2012. As shown in the 10 Results section below, we abandoned the use of the *BMC Public Health* protocols as the source of prespecified outcomes since so few were published before the start times of the studies they described. Instead, we used the registries cited in these protocols as the source of the prespecified outcomes. However, since half of the registrations were also retrospective, we had to expand the timeframe from 2011 to 2012 to 2011-2015 in order to identify a sufficient number of protocols that described studies that had been prospectively registered.

In the registry, we proposed examining the following discrepancies between the protocols and publications: (a) a primary outcome in the protocol is reported as a secondary outcome in the published paper; (b) a secondary outcome in the protocol is reported as a primary outcome in the published paper; (c) a primary outcome described in the protocol is omitted in the published paper; (d) a secondary outcome described in the protocol is omitted in the published paper; (e) a primary outcome that is not described in the protocol is reported in the published paper; (f) a secondary outcome that is not described in the protocol is reported in the published paper. Given the additional time and work required to collect data from registries in order to conduct the study, we limited the analysis to primary outcomes reported in these and therefore did not assess the concordance between registries and publications on secondary outcomes.

## Results

**Fig. 1:**
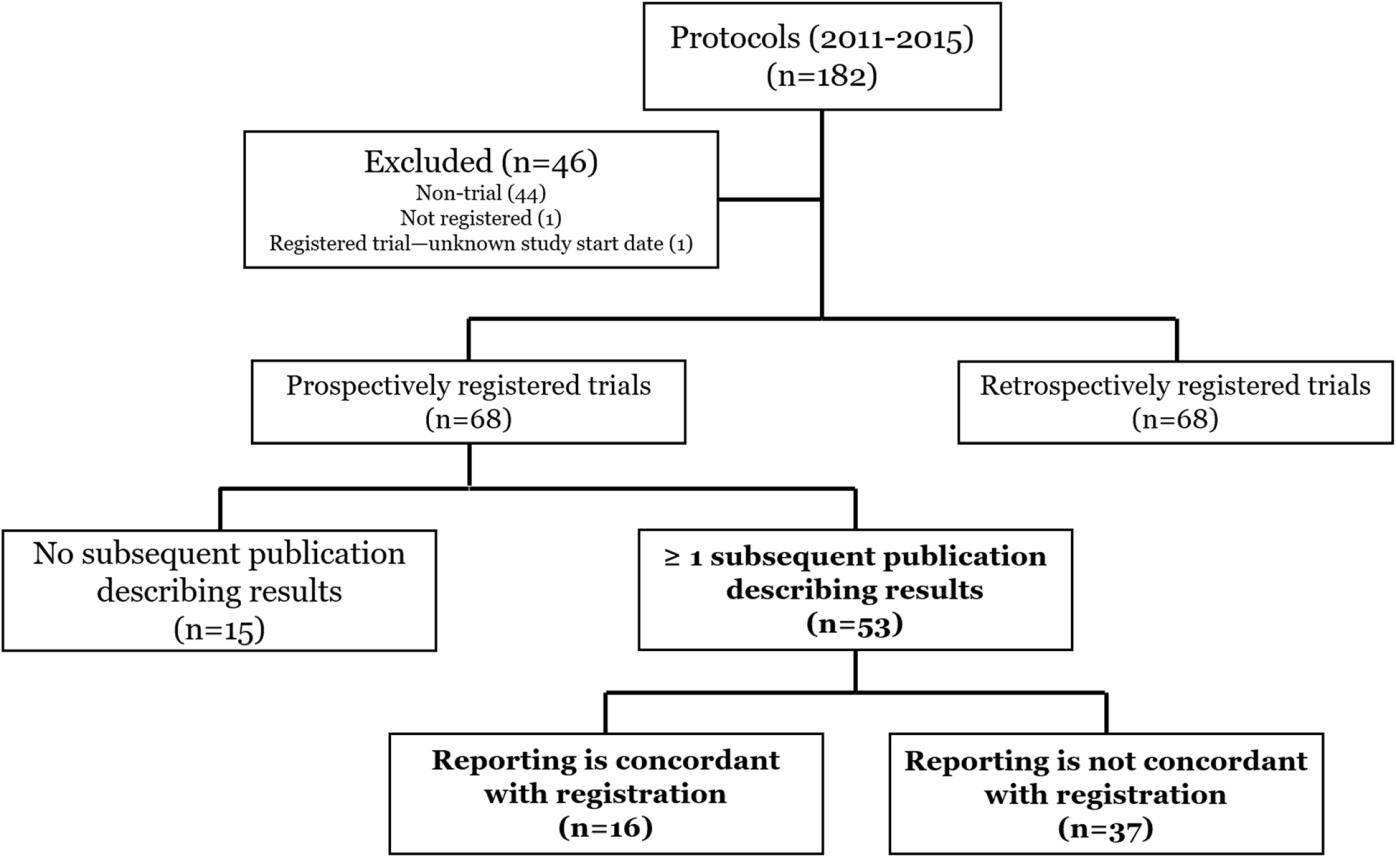
Flow chart illustrating protocols identified in the Health Behavior, Health Promotion and Society section of BMC Public Health between 2011 and 2015, as well as the subsequent adjudication of RRCTs and publications included in the analysis

Figure 1 shows the protocols identified in the *Health Behavior, Health Promotion and Society* section of *BMC Public Health* between 2011 and 2015, as well as the subsequent adjudication of RRCTs and publications included in the analysis. There were 182 study protocols published between 2011 and 2015; 46 protocols (25%) were excluded from analysis because: they did not describe a randomized trial, the registry entry could not be found for a purported registered trial, or the registry entry did not contain a trial start date. The remaining 136 RRCTs comprised the final analytic cohort.

Table 1 shows that 68 of 136 trials within the analytic cohort were prospectively registered. However, only 16 of the protocols describing these studies were submitted to *BMC Public Health* before the reported start date of the trial, and just 15 were both prospectively registered and associated with a prospectively submitted protocol. Among the 120 protocols published after the trial began, there was an average period of approximately 21 months (range: 0.2-79.2) between commencement of the trial and submission of the protocol for publication in *BMC Public Health*. Moreover, 77 of those 120 protocols were submitted to the journal 12 or more months after the associated trials began.

**Table 1:** Trial registration type according to protocol submission type

		Registration Type		
		Prospective	Retrospective	Total
**Protocol Submission Type**^**a**^	Prospective	15	1	16
	Retrospective	53	67	120
	Total	68	68	

^a^Prospectively submitted protocols are those published before commencement of the associated trial

The 68 prospectively registered trials were the focus of the next phase of the study—assessing agreement between the outcomes reported in the registry and those reported in subsequent published journal articles. The combined registry search, database search and investigator-replies to e-mails (a response was received from 54 of 66 sent; one investigator was deceased and one did not have a current e-mail address) led to the identification of at least one published article reporting primary outcomes for 53 of the 68 prospective RRCTs. Of the 15 trials for which we could not identify a published manuscript describing results, data collection was still underway for one trial [25]. Relevant primary outcome results were available in a single manuscript for 45 of these RRCTs, with the remaining eight publishing primary outcome results in two manuscripts.

Among the 53 prospective RRCTs for which at least one published paper was identified, only 16 (30%) contained information concordant with the subsequent reporting of published results (Table 2). The 37 prospective RRCTs containing information that was not consistent with published trial results were discrepant in eight key ways highlighted in Table 2, and 67% (n = 25) of those discordant studies contained more than one discrepancy.

**Table 2:** Registry-publication concordance and discrepancies in discordant studies

Studies	n (***%***)
**Concordant vs. discordant studies (*n*=53)**	
Concordant	16 (*30*)
Discordant	37 (*70*)
**Discrepancies identified in discordant studies (*n*=37)**	
Primary outcome(s) described in registration was/were not explicitly defined	19 (*36*)
Registration did not state how primary outcome(s) would be measured/codified	16 (*30*)
Published manuscript(s) reporting results of registered trial include at least one unregistered primary outcome	16 (*30*)
Primary outcome(s) described in registration was/were not measured according to registration prescribed timing protocol	14 (*26*)
Published manuscript(s) reporting results of registered trial did not report on all registered primary outcomes	11 (*21*)
Secondary outcome(s) described in registration was/were promoted to primary outcome(s) in published manuscript(s) reporting trial results	5 (*9*)
Registration prescribed timing protocol for measuring primary outcome(s) was not adequately defined	4 (*7*)
Primary outcome(s) described in registration was/were de-emphasized or demoted to secondary outcome(s) in published manuscript(s) reporting trial results	4 (*7*)

Two of the top three most commonly identified discrepancies involved vague descriptions of the primary outcomes in the registry and failure to specify how these would be measured, whereas the publications contained detailed descriptions of the outcomes and specific measurement instruments. There was considerable overlap of these two features, with 15 of the 16 studies that failed to specify measures in the registry also describing the primary outcomes in a vague manner.

It was more common for unregistered primary outcomes to be added to publications than for registered primary outcomes to be excluded from publication of trial results. Specifically, 16 of the studies introduced a primary outcome into the publication that was not registered at the start of the study, although four of these subsequently amended the registration to include these new primary outcomes. Three of these studies, along with two others, promoted a registered secondary outcome to a primary outcome in the publication. In contrast, 15 of the studies either failed to report a registered primary outcome (*n* = 11) or relegated it to a secondary outcome in the publication (*n* = 4), with no overlap in these categories.

Regarding adherence to the schedule of follow-up assessments in these publications: registry entries for three studies contained poorly defined timing protocols, 13 failed to adhere to the registered protocol, and one timing protocol was both poorly defined and not adhered to in the publication.

On average, the 37 discordant studies displayed 2.4 of the eight registry-publication discrepancies pertaining to either primary outcomes and/or assessment protocols described in the registry in the publications from the study.

## Discussion

The current study examined the extent to which registration of behavioral and health promotion trials published in *BMC Public Health* reduced selective outcome reporting. A fundamental precondition of this is that the trial be registered prospectively, that is, before commencement of data collection. As is the case with registration of clinical trials in medical research, we found that half of the registered behavioral and health promotion trials examined were registered retrospectively [16–18]. We also found that the vast majority of the published protocols associated with these trials were submitted after the start of the trial. While retrospective protocol publication does not undermine reproducibility studies, this practice is not in accord with SPIRIT guidelines and fundamentally undermines *BMC Public Health*’s goal of reducing publication bias, since such protocols can incorporate *post hoc* modifications of variable definitions and analytic methods.

Moreover, retrospective registration of trials may actually corrupt the research literature in a manner that is worse than non-registration. Registration gives a study greater credibility, as it is assumed the investigators are reporting results from a pre-specified analysis plan with pre-specified outcomes [26]. At the time the reviewed protocols were published, *BMC Public Health* included a date in each registration, but it was unclear whether this date preceded the start date of the trial; the journal has since changed its submission guidelines and requires trials that were not registered before enrollment of the first participant to include the words “retrospectively registered” in submitted manuscripts [27]. The Authors believe this is a very good policy, and one that ClinicalTrials.gov should adopt.

Registration documents are also used by the Cochrane Systematic Reviews in their assessment of selective outcome reporting bias [28]. Specifically, Cochrane strongly encourages “review authors to attempt to retrieve the pre-specified analysis intentions for each trial” and states that the best sources of such information are trial registry entries and trial protocol or design papers published in journals.” Only documents that are date-stamped “confirming the analysis intentions were finalized before unblinded outcome data were available to trial investigators” should be used by reviewers in assessing risk of bias resulting from selective reporting. Recent evidence shows that Cochrane reviewers perform selective outcome reporting bias assessments very poorly and appear to make little use of registries and published protocols [29]. The reasons for this are not entirely clear, but the current research suggests that there is a large proportion of registries (about half) and published protocols (~90%) that do not meet the requirements set by Cochrane for assessing selective outcome reporting. Indeed, it would be a problem if these retrospectively produced registries and protocols were used to assess selective outcome reporting bias, since low bias might simply reflect the fact that the outcomes reported in these were determined after data were collected and analyzed.

Similar to studies of selective outcome reporting in registered clinical trials, we found that about a third of registered behavioral and health promotion trials continue to fail to report all registered primary outcomes or demote them to secondary outcomes and about one in four trials introduce an unregistered primary outcome into subsequent publications. The timing of follow-up assessments were also not followed in nearly 40% of studies. Our review also allowed us to assess the quality of the registry entries, specifically the precision with which outcomes were defined and the extent to which details of the measures to be used were included in the registry entry; over half contained vague definitions and 40% did not state how the outcome would be measured.

Lack of specificity in outcome measures may be a greater problem in behavioral trials than drug and medical trials, as the latter are subject to regulatory oversight by government agencies (e.g. the Food and Drug Administration in the USA) and are likely to have specific clinical outcomes that will be closely monitored according to a set of trial “stopping rules”. In contrast, behavioral trials typically investigate less-specific outcomes, such as: diet adherence or compliance, physical activity, sedentary behavior, abstinence from drug use and binge drinking. The manner in which outcomes of this nature can be defined and measured is diverse, and a registry is of limited value if an accompanying measurement instrument and/or procedure is not detailed.

There are a few limitations that should be noted. First, the analysis was limited to trials that published a protocol in the *Health Behavior, Health Promotion, and Society* section of *BMC Public Health*. It is possible that behavior and promotion health trials that published their protocols in other journals or other sections of *BMC Public Health* displayed different levels of concordance between registry entries pertaining to outcome variables and outcomes reported in subsequent publications. Second, although we used a number of different procedures to identify publications from each trial, it is possible that some trial authors published results subsequent to completion of our review or that we failed to identify eligible publications. Third, registries and publications were reviewed by just two individuals and it is possible that different reviewers could have come to different conclusions about concordance with respect to primary outcome variables reported in registration submission and subsequent publication of trial results. Fourth, the sample of studies examined was selected on the basis of them having published a protocol in *BMC Public Health* between 2011 and 2015 in order to allow sufficient time for them to conclude and for the investigators to produce publications reporting results. It is possible that the timeliness of protocol publication and registration in relation to the start of a study may have improved more recently, as might adherence to protocols and registered primary outcomes in subsequent publications. Fifth, we used a cut-off of 10 days from the start of each study to determine whether it was registered retrospectively or prospectively. It might be argued that this is an excessively strict time-frame as administrative or logistical reasons might delay submission of materials to a trial registration site. However, one of the most important reasons for registering a trial is to indicate the *a priori* consideration of study design and conduct.

## Conclusions

In conclusion, our study indicates that the registration of behavioral and health promotion trials displays many of the same limitations found in its application within medical research more generally. Half of these trials were retrospectively registered, and prospectively registered trials frequently added unregistered outcomes to publications, failed to report registered outcomes, and described outcomes and their measurement in a vague manner in the registry. These problems, which also pervade the clinical trials literature, are likely the result of a lack of understanding of the central purpose of registration (i.e., to reduce selective outcome reporting bias) by many investigators in the social and behavioral sciences. The problem is compounded by the allowance of widespread retrospective registration and unfettered investigator modification of registration entries. Incomplete and vague registry entries in key study design features such as the description of primary outcome variables also means that registries will have limited utility in terms of facilitating replication studies. Published protocols, such as those from *BMC Public Health* that were the starting point of the current study, might be more useful since they are typically much more detailed than registration submissions. However, because so many of these are retrospectively submitted for publication, they are of little use in preventing selective outcome reporting.

We recommend that, moving forward, registries no longer accept retrospective registrations; in practice, this would probably mean phasing them out over a period of two or three years. For existing registrations, there should be a clear differentiation on the registry web page of those that were prospectively registered and those that were retrospectively registered. Ideally, there should be a program officer or administrator who reviews and has oversight of submissions and any changes that occur to fields such as primary and secondary outcomes. Journals should also clearly differentiate retrospective from prospective registration in the manuscripts they publish. With regard to protocols, journals should also require authors to specify when they were written in relation to the timelines of the studies they describe. Only those written before the study begins can be used to assess the consistency and rigor of trial conduct when compared to subsequent publications. Those written after a study has begun are essentially extended descriptions of the study methods, and might be more appropriately included as appendices in publications reporting results rather than published as stand-alone "protocols".

We consider the most important reason for registering a trial to be the *a priori* indication of study design, conduct, and analyses. As noted by Wagenmakers et al. [4], only such preregistered studies warrant the designation “confirmatory” and while *post hoc* analyses are perfectly legitimate, they must be clearly labeled “exploratory”. Journals can easily make such distinctions among papers they publish.

## Supplementary Information

**Additional file 1.**

## Data Availability

All data generated or analyzed during this study are publicly available and are also included in this published article [and its supplementary information files].
